# Cecal Volvulus Through an Internal Hernia Created by an Elongated Fallopian Tube

**DOI:** 10.7759/cureus.34943

**Published:** 2023-02-13

**Authors:** Maianh Tran, Justin K Song, John Popovich

**Affiliations:** 1 General Surgery, Mercy Medical Center, Des Moines, USA; 2 General Surgery, Des Moines University's College of Osteopathic Medicine, Des Moines, USA

**Keywords:** strangulated hernia, cecal volvulus, large bowel obstruction, elongated fallopian tube, internal hernia

## Abstract

Internal hernias result from abdominal viscera protruding through a congenital or acquired defect in the peritoneum or the mesentery of the abdominal cavity. They are less common than external hernias, and the overall incidence is rare. Internal hernias carry a high mortality rate if there is no immediate surgical intervention and can lead to complications such as bowel perforation, ischemia, and necrosis. There are multiple classifications, and a rare subtype identified in only a select few cases involves the fallopian tube. This case documents the development of a cecal volvulus due to the cecum herniating through an aperture created by a normal-appearing fallopian tube attaching to the retroperitoneum.

A 78-year-old female with multiple comorbidities was admitted for abdominal pain lasting 3-4 days, nausea, emesis, and poor oral tolerance. Computerized tomography imaging revealed a complete large bowel obstruction secondary to a cecal volvulus, and she was taken emergently for an exploratory laparotomy. Intra-operatively, a distended cecum was noted, herniated through a loop created by the right fallopian tube tethering its free end to the left pelvis. Upon decompression of the bowel, the fallopian tube released itself from the retroperitoneum. The cecum and right fallopian tube were noted to be ischemic and resected with an ileo-transverse anastomosis. Internal hernias that involve the fallopian tubes are a rare variation of an already uncommon condition. However, they should be included in the differential diagnosis when evaluating a female patient for intestinal obstruction since it can develop into a life-threatening condition that requires prompt surgical attention.

## Introduction

An internal hernia is an uncommon cause of intestinal obstruction with an incidence of < 1% [1]. It can become life-threatening with a high mortality rate of > 50% if intra-abdominal contents are strangulated and the condition is left untreated [2]. The most common type of internal hernia is the paraduodenal (53%), followed by pericecal (13%), foramen of Winslow (8%), transmesenteric and transmesocolic (8%), intersigmoid (6%), and retroanastomotic (5%) [1]. There is also an increased incidence of acquired internal hernia as more patients undergo surgical procedures such as gastric bypasses with Roux-en-Y reconstruction [3]. However, few documented cases of internal hernias involve the fallopian tube. This case demonstrates a rare instance where the fallopian tube was attached to the retroperitoneum creating a window for a mobile cecum to herniate and form a volvulus.

## Case presentation

**Figure 1:**
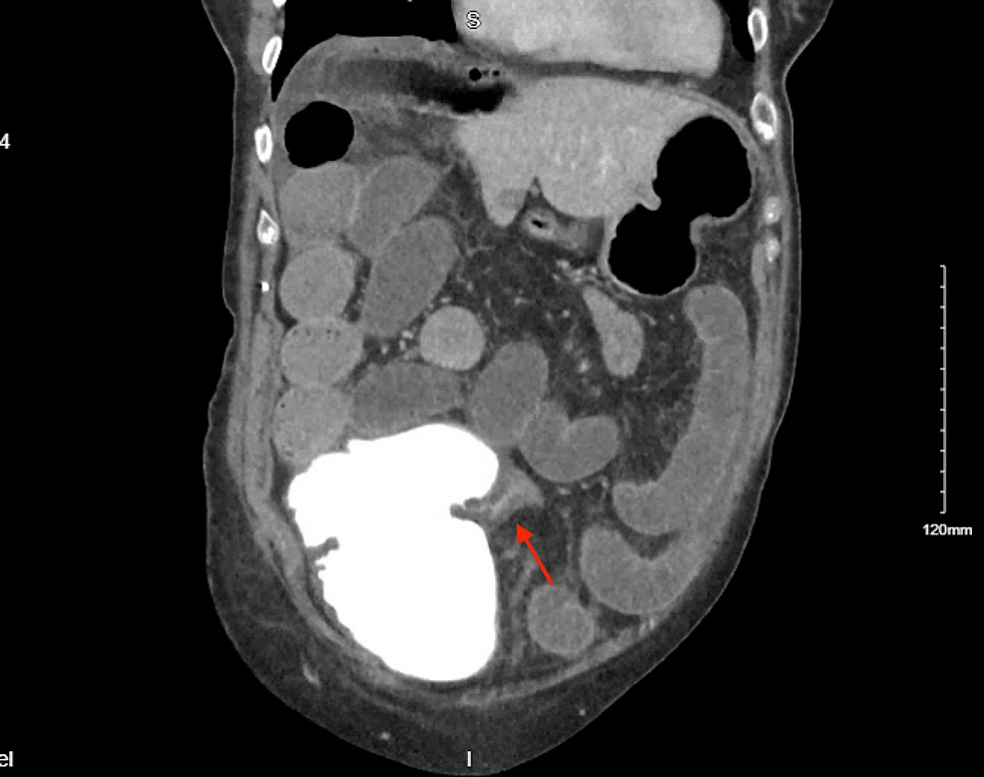
Coronal View of Terminal ileum (red arrow) Entering Cecum

**Figure 2:**
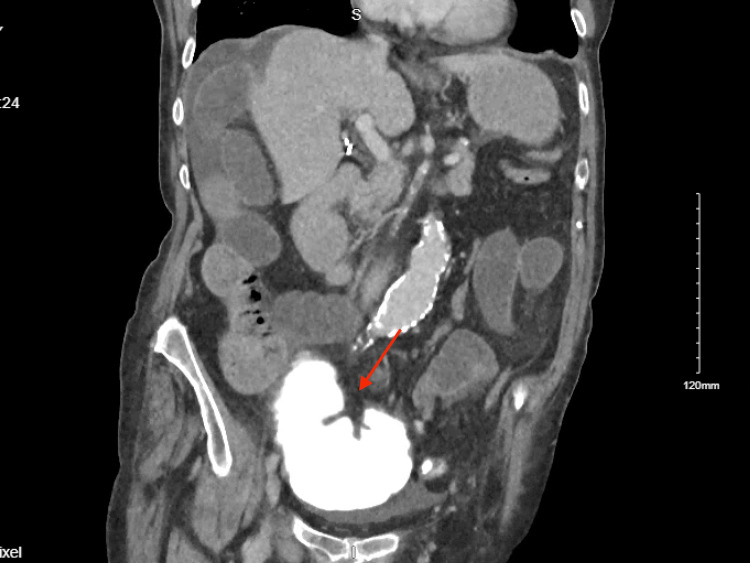
Internal Herniation of Cecum with no Distal Contrast

The patient is a 78-year-old female who presented with 6 days of abdominal pain, nausea, vomiting, poor oral tolerance, belching, fevers, chills, and sweats. In the week prior, she was seen multiple times in the Emergency Department for similar symptoms. However, imaging at these visits showed benign radiologic findings with no explanation for her pain. She had been eating poorly for several months prior to her symptom onset due to dysphagia associated with iatrogenic vocal cord paralysis. She lost approximately 30-40 pounds while maintaining a liquid-supplemented diet. Her last colonoscopy was 10 years prior and was reported to be normal with no indicated follow-up. A computerized tomography scan of the abdomen and pelvis with intravenous and oral contrast showed multiple dilated loops of the small bowel and cecum (Figure 1), with the contrast failing to pass through to the decompressed distal colon (Figure 2). With concerns for a suspected large bowel obstruction secondary to an internal hernia, she was transferred to a tertiary hospital and underwent an exploratory laparotomy.

**Figure 3:**
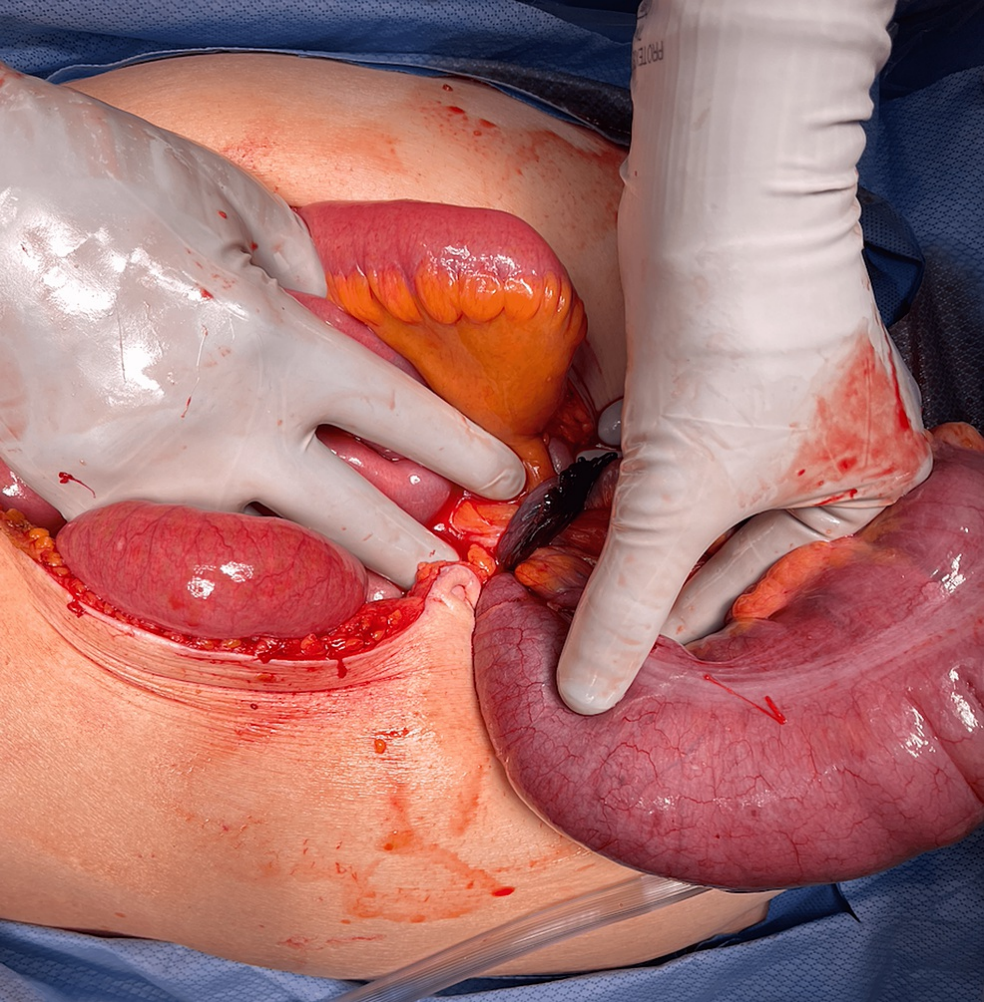
Herniated Cecum through an Orifice Created by the Right Fallopian Tube

The patient agreed to surgical intervention and was given Cefoxitin pre-operatively. Upon intra-abdominal access, there were murky ascites with several distended bowel loops. Cultures were taken and ultimately negative for aerobes, anaerobes, and acid-fast bacteria. A mobile cecum was noted herniating through a defect created by the right fallopian tube, which had twisted upon itself (Figure 3). Ischemic changes were seen in the strangulated cecum, right fallopian tube, mid-transverse colon, and distal 30 cm of the ileum. A controlled colotomy was performed to decompress and reduce the colon. Following this, the fallopian tube spontaneously detached from the left pelvis. The ischemic tissue was resected to include the terminal ileum, ascending colon, proximal transverse colon, and a right salpingo-ophorectomy. The remaining bowel was inspected before performing an ileocolonic anastomosis. The specimens sent to pathology were all found to be histologically unremarkable. The patient’s postoperative period was overall uneventful. She continued on Zosyn post-operatively for five days and was discharged on hospital day five after diet tolerance and meaningful return of bowel function.

She followed up with the Emergency General Surgery clinic two weeks after discharge and was noted to be healing well with minor complaints of diarrhea. Education on maintaining a high-fiber diet was provided along with as-needed use of Loperamide. She continues to see her Primary Care Physician and has been recovering since her operation.

## Discussion

The patient discussed in this report is a 78-year-old female who was transferred for symptoms of intestinal obstruction. The intra-operative investigation demonstrated an internal herniation of the cecum through an atypical orifice created by the right fallopian tube attaching to the retroperitoneum. This unusual case was made possible by combining two equally rare features noted intra-operatively. First, the patient’s cecum was found to have very limited retroperitoneal connections that enabled it to be mobile and herniated. Second, the distal end of the right fallopian tube was anchored and attached to the retroperitoneum in a way that permitted colonic herniation. 

A cecal volvulus is an uncommon cause of bowel obstruction, with an incidence rate of less than one percent in all adult cases [4]. It can occur when a mobile portion of the proximal colon twists upon itself using a fixed point of the bowel as a fulcrum. There are several precipitating factors to forming a cecal volvulus, including recent abdominal surgeries, adhesions, and masses [4]. An essential prerequisite is the presence of a mobile cecum, which is estimated to occur congenitally in 10-25% of the population [5]. This condition typically arises when the normal peritoneal attachments fail to form due to disruption or over-rotation of the proximal colon during fetal development [4].

Internal hernias are rare causes of intestinal obstruction, and it is even more uncommon for the fallopian tube to be involved in its formation. One case reports a small bowel strangulation secondary to an internal hernia caused by a defect in the broad ligament and a dilated left fallopian tube wrapping around the bowel to mimic a band adhesion [6]. Another case reports a strangulated bowel caused by an internal herniation through a peritoneal defect between the right fallopian tube and ovarian vessels [2]. A third case describes an incarcerated mobile cecum resulting from an internal herniation through a loop created by an elongated left fallopian tube of 25 cm [5]. While the patient illustrated in this case presents similarly to our patient, our patient’s right fallopian tube was 9.5 cm in length by 0.7 cm in diameter, which is closer to the average length of fallopian tubes seen in females in their 70s of 8.8 cm [7]. The distal portion of the right fallopian tube was found with atypical adhesions that connected it to the retroperitoneum. While the etiology behind the adhesions is unknown at this time, these adhesions anchored an otherwise unremarkable fallopian tube to the peritoneum in such a way that it created the ideal passageway for mobile to cecum to herniate through. 

Internal hernias are a severe condition that requires immediate surgical attention as they may lead to bowel ischemia, necrosis, and eventual perforation if left untreated. This case report details a pathologically normal fallopian tube strangulating a mobile cecum. The exact origin of the adhesions connecting the fallopian tube to the retroperitoneum remains unclear. However, this case shows the importance of considering the fallopian tube when treating a female patient for an internal hernia.

## Conclusions

Very few cases have documented internal hernias that involve the fallopian tube. This patient presented with a highly unusual situation where the combination of two anatomical anomalies resulted in the strangulation of the cecum and ischemic changes to the neighboring bowel: a mobile cecum and fallopian tube of age-appropriate length attached to the retroperitoneum. Involvement of the fallopian tube should be considered in the differential when diagnosing a female patient without a history of salpingo-oophorectomy for an internal hernia. While rare, congenital or acquired changes to the fallopian tube may increase the risk of a hernia.
